# A KDM5 variant outside the catalytic Jumonji C domain disrupts demethylase activity and alters the proximity interactome

**DOI:** 10.1093/g3journal/jkag219

**Published:** 2026-08-06

**Authors:** Melissa A Castiglione, Matanel Yheskel, Aubrey A Siebels, Simone Sidoli, Julie Secombe

**Affiliations:** Department of Genetics, Albert Einstein College of Medicine, Bronx, NY 10461, United States; Department of Genetics, Albert Einstein College of Medicine, Bronx, NY 10461, United States; Dominick P. Purpura Department of Neuroscience, Albert Einstein College of Medicine, Bronx, NY 10461, United States; Department of Biochemistry, Albert Einstein College of Medicine, Bronx, NY 10461, United States; Department of Genetics, Albert Einstein College of Medicine, Bronx, NY 10461, United States; Dominick P. Purpura Department of Neuroscience, Albert Einstein College of Medicine, Bronx, NY 10461, United States

**Keywords:** KDM5, KDM5C, histone demethylase, chromatin, transcription, Claes–Jensen syndrome, KDM5C-NDD

## Abstract

Transcriptional programs regulated by the KDM5 family of chromatin-modifying proteins are dysregulated in cancer and intellectual disability (ID) disorders. To define the fundamental mechanisms by which KDM5 regulates disease-relevant gene expression, we use missense variants in the X-linked KDM5C gene associated with the ID disorder Claes–Jensen syndrome (also known as KDM5C-NDD). Here, we use *Drosophila melanogaster* to investigate the effects of KDM5^A224T^, equivalent to human KDM5C^A77T^, which affects a conserved residue outside the catalytic histone demethylase JmjC domain and alters both enzymatic and non-enzymatic activities. Quantifying levels of H3K4me3, the demethylase substrate of KDM5, in adult brains revealed that *Kdm5^A224T^* induced changes indistinguishable from those observed with a catalytically inactive allele. This effect was not due to reduced promoter recruitment of the variant KDM5^A224T^ protein. Instead, TurboID studies demonstrate that KDM5^A224T^ exhibits reduced proximity with proteins involved in promoter activity and chromatin remodeling. Together, these findings show that KDM5-dependent transcriptional regulation cannot be explained by demethylase activity alone and support altered chromatin regulatory interactions as a key mechanism underlying pathogenic KDM5 variants.

## Introduction

Post-translational modifications of histone proteins within chromatin play central roles in regulating gene expression (Allis and Jenuwein 2016). Accordingly, the enzymes that deposit, interpret, and remove these marks are essential for proper transcriptional control (Hyun et al. 2017; Shvedunova and Akhtar 2022). The lysine demethylase 5 (KDM5) family of proteins comprises 4 paralogs in mammals (KDM5A–KDM5D) and a single ortholog in *Drosophila melanogaster*. Consistent with their fundamental role in gene regulation, altered expression of all 4 KDM5 proteins has been linked to a wide range of cancers (Chen et al. 2025). In addition, disruption of KDM5A, KDM5B, or KDM5C function can lead to neurodevelopmental disorders characterized by changes to cognition, including intellectual disability (ID), seizures, and other behavioral changes (Goncalves et al. 2014; Carmignac et al. 2020; Hatch and Secombe 2022; Leonardi et al. 2023; Ghasemi et al. 2025; Terry et al. 2026). Notably, variants associated with cognitive dysfunction have not been linked to increased cancer risk, highlighting a gap in our understanding of how distinct KDM5 activities contribute to diverse disease outcomes (Terry et al. 2026).

Across species, KDM5 proteins demethylate histone H3 trimethylated at lysine 4 (H3K4me3) through the joint activity of their Jumonji N (JmjN) and Jumonji C (JmjC) domains (Holowatyj et al. 2015; Pavlenko et al. 2022). H3K4me3 is primarily observed near promoters of actively transcribed genes, is recognized by numerous gene-regulatory proteins, and plays key roles in transcriptional consistency (Wang et al. 2023; Yu and Lesch 2024). In addition to their enzymatic demethylase function, KDM5 proteins have additional domains that impart other functions that are likely to influence gene expression in concert with, or independently of, the JmjN/JmjC domains. These include plant homeodomain (PHD) motifs that bind to the tail region of histone H3, an AT-rich interaction domain (ARID) that binds to DNA in vitro, and others whose activities are not yet defined (Tu et al. 2008; Wang et al. 2009; Li et al. 2010; Yao et al. 2010; Klein et al. 2014; Peng et al. 2015). Integral to understanding the role of KDM5 proteins in human disorders is defining how both individual and combinatorial domain activities function to regulate gene expression.

Missense variants associated with ID provide a powerful approach to dissect KDM5 function, as they highlight residues critical for protein activity. Approximately half of the ∼122 pathogenic variants identified in the X-linked *KDM5C* gene are missense alleles, including many within the JmjC domain (Carmignac et al. 2020; Ghasemi et al. 2025; Terry et al. 2026). This enrichment is consistent with evidence from in vitro and in vivo studies supporting an important role for the histone demethylase activity of KDM5C in driving patient phenotypes. In vitro, several missense variants in human KDM5C reduce enzymatic activity (Iwase et al. 2007; Tahiliani et al. 2007; Brookes et al. 2015). In vivo, a demethylase-dead allele that disrupts Fe^2+^ coordination within the JmjC domain in *Drosophila* KDM5 (H637A and E639A; Kdm5^JmjC*^) alters synaptic activity and movement in larvae, in addition to deficits in learning and locomotion in adults (Zamurrad et al. 2018; Belalcazar et al. 2021; Yheskel et al. 2024). Consistent with this, some behavioral changes in *Kdm5C* knockout mice can be partially rescued by restoring H3K4me3 levels (Vallianatos et al. 2020).

In addition to their canonical histone demethylase activity, KDM5 family proteins possess non-enzymatic gene-regulatory functions that are increasingly recognized as important contributors to both cancer and ID (Li et al. 2010; Hatch and Secombe 2022; Di Nisio et al. 2024). Using *Drosophila*, we previously generated 6 *Kdm5* alleles analogous to patient ID variants and demonstrated that they produce a spectrum of cognitive and behavioral phenotypes broadly consistent with traits observed in humans (Zamurrad et al. 2018; Belalcazar et al. 2021; Yheskel et al. 2024). Among these, *Kdm5^A224T^*, equivalent to the ID-associated *KDM5C^A77T^* allele, is particularly informative because it exhibits features suggesting it affects both demethylase-dependent and independent activities of KDM5. *Kdm5^A224T^* flies display a 2-fold increase in bulk H3K4me3 comparable to that observed in *Kdm5^Δ^* null allele and demethylase-dead Kdm5^JmjC*^ animals, indicating loss of histone demethylase activity (Drelon et al. 2018; Zamurrad et al. 2018; Yheskel et al. 2024). However, *Kdm5^A224T^* causes axonal growth and guidance defects similar to those observed in *Kdm5^Δ^* null-allele animals, but that are absent in Kdm5^JmjC*^, indicating disruption to non-enzymatic functions of KDM5 (Hatch et al. 2021; Yheskel et al. 2024). These phenotypes could not be attributed to loss of KDM5 protein, as KDM5^A224T^ is expressed at wild-type levels, localizes to the nucleus, and supports viability to adulthood, unlike *Kdm5^Δ^* animals that die during the pupal stage of development (Drelon et al. 2018, 2019; Yheskel et al. 2024). This allele, therefore, provides a unique opportunity to gain insights into demethylase-dependent and independent mechanisms of KDM5-mediated transcriptional regulation.

Here, we exploit the pathogenic *Kdm5^A224T^* allele as a genetic tool to investigate how KDM5 regulates gene expression. Quantification of H3K4me3 in adult brains demonstrated that KDM5^A224T^ behaves functionally as a demethylase-dead protein. In addition, re-analysis of previously generated RNA-seq data revealed gene expression deficits shared with Kdm5^JmjC*^ animals, as well as genes uniquely affected by *Kdm5^A224T^*, indicating defects in both enzymatic and non-enzymatic KDM5 activities (Zamurrad et al. 2018; Yheskel et al. 2024). Despite the proximity of A224 to the putative nucleic acid-binding ARID, the KDM5^A224T^ protein does not substantially alter the ability of KDM5 to be recruited to its genomic sites. Instead, KDM5^A224T^-induced alterations in protein–protein interactions are likely to contribute to these additional gene expression deficits, as the TurboID proximitome of this variant protein showed significant changes to many chromatin and transcriptional regulators. Notably, a subset of these proximity changes was shared by KDM5^JmjC*^, suggesting a link between enzymatic activity and complex formation. Together, our results demonstrate that regions outside of the catalytic JmjN/JmjC domains are critical for KDM5 function and highlight the importance of protein interactions in both demethylase-dependent and independent regulatory pathways. These findings provide new insight into the molecular basis of KDM5-associated neurodevelopmental disorders.

## Materials and methods

### Resource availability

Further information and requests for reagents should be directed to Julie Secombe (julie.secombe@einsteinmed.edu).

### Fly strains and care

Fly food (per liter) contained 80 g malt extract, 65 g cornmeal, 22 g molasses, 18 g yeast, 9 g agar, 2.3 g methyl para-benzoic acid and 6.35 mL propionic acid per liter. Unless otherwise stated, flies were kept at 25 °C with a 12-h light/dark cycle and 50% humidity. All flies were collected or harvested 1 to 5 d after eclosion between 9 am and 12 pm. *Kdm5*^Δ^ (null allele; previously *Kdm5^140^*), *Kdm5^A224T^* and Kdm5^JmjC*^ are published (Zamurrad et al. 2018; Belalcazar et al. 2021; Yheskel et al. 2024). UASp-*TurboID*:*Kdm5*^A224T^ and *TurboID*:Kdm5^JmjC*^ constructs were generated by site-directed mutagenesis of published UASp-*TurboID*:*Kdm5,* and transgenes were inserted into the attP site at 86F (Yheskel et al. 2023). The UAS-*Dam*:*Kdm5*^A224T^ variant construct was generated by site-directed mutagenesis of the published UAS*-Dam*:*Kdm5* (Hatch et al. 2021; Rogers et al. 2023). All Dam fusion transgenes were inserted into attP2 on chromosome III. All transgenesis was carried out at The Best Gene. The Ubi-Gal4 fly strain (RRID:BDSC_32551) is publicly available through the Bloomington *Drosophila* Stock Center.

### Antibodies and western blot analyses

The anti-KDM5 antibody has been previously validated and published (Secombe et al. 2007). Anti-H3K4me3 was from Epicypher Cat# 13-0041 (RRID:AB_3076423), and anti-Tubulin was from Cell Signaling Technology Cat# 2144 (RRID:AB_2210548). For western blots, five 2- to 5-d-old adult heads were homogenized in 2× NuPAGE LDS sample buffer, sonicated for 10 mins, treated with DTT, run on a 4 to 12% Bis-Tris 1 mm gel, and transferred to a PVDF membrane. Primary antibodies used were as follows: Rabbit anti-KDM5 (1:100), Streptavidin 680 (1:10,000, ThermoFisher, Streptavidin Alexa Fluor 680 conjugate), rabbit anti-alpha-Tubulin (1:5,000). Secondary antibodies used were IRDye 680RD Donkey anti-Mouse IgG (1:5000; LI-COR Biosciences Cat# 925-68072, RRID: AB_2814912) and IRDye 800CW Donkey anti-Rabbit IgG (1:5,000; LI-COR Biosciences Cat# 926-32213, RRID: AB_621848). Blots were scanned and processed using a LI-COR Odyssey Infrared scanner.

### Structural modeling

Structural models of *Drosophila melanogaster* KDM5 (previously known as Lid) were derived using AlphaFold (Jumper et al. 2021). For KDM5^WT^, KDM5^A224T^, and KDM5^JmjC*^ (H637A/E640A), 5 independent AlphaFold models were used to account for model variability. All analyses were performed on full-length protein models using consistent residue numbering across conditions. Residue interaction networks (RINs) were generated for each AlphaFold model using RING 4.0 (Residue Interaction Network Generator) (Del Conte et al. 2024). Interactions between residue pairs were defined using standard geometric criteria and classified by type (hydrogen bonds, van der Waals contacts, ionic interactions, π–π stacking, cation–π interactions, and π–hydrogen bonds). Analyses were restricted to strong interactions: hydrogen bonds, ionic interactions, π–π stacking, cation–π interactions, and π–hydrogen bonds, but excluding van der Waals–only contacts. Edges were treated as undirected and stored as canonicalized residue pairs with interaction type. Similarity between consensus RINs was quantified using the Jaccard similarity coefficient, defined as the size of the intersection divided by the size of the union of 2 edge sets. Relative to WT, edges were classified as lost (present in WT consensus but absent in variant consensus) or gained (absent in WT consensus but present in variant consensus).

To assess whether loss of enzymatic activity correlated with disruption of the catalytic site, a pocket-localized RIN analysis was performed. The catalytic pocket was defined geometrically using the known Fe^2+^-binding residues of KDM5. Residues H637, E640, and H725 were used as anchor residues. All residues with any atom within 8 Å of any anchor residue were classified as pocket residues. Consensus RINs were filtered to include only edges for which at least one residue belonged to the catalytic pocket. For each mutant, pocket-localized networks were compared to WT using 2 complementary metrics: pocket edge retention, defined as the fraction of WT pocket edges preserved in the mutant network, and pocket similarity, defined as the Jaccard similarity between WT and mutant pocket edge sets.

Hydrogen bonding interactions involving the residue of interest were examined using UCSF ChimeraX (Pettersen et al. 2004, 2021). Wild-type and mutant protein structures were loaded into ChimeraX, and hydrogen bonds were identified using the FindHBond tool with default geometric criteria. The local interaction networks surrounding the residue of interest were visually compared between wild-type and mutant structures to identify hydrogen bonds that were retained, lost, or newly formed following amino acid substitution.

### Targeted DamID (TaDa)

Tissue processing was performed as previously described, with the following modifications: TaDa was performed in triplicate for each genotype, using 10 heads per sample (Hatch et al. 2021; Yheskel et al. 2024). These TaDa experiments were performed in parallel to those reported previously, allowing for direct comparisons between Dam:KDM5^A224T^ and Dam:KDM5^JmjC*^ data sets (Yheskel et al. 2025). Dam:KDM5 fusion proteins were expressed under the control of Ubi-Gal4, which was pulsed on for 3 d during early adulthood. Genotypes were *Kdm5^Δ^*, Ubi-Gal4/*Kdm5^Δ^*, tubulin-Gal80^ts^; UAS-*Dam:Kdm5^WT^* or variant, *gKdm5:HA^WT or variant^*. Samples were homogenized in 75 μl UltraPure Distilled Water and 20 μl 500 mM EDTA then digested with Proteinase K for 1.5 h. DNA extraction was performed using the Zymo Quick-DNA Miniprep Plus Kit (Zymo, D3024). DpnI digestion, PCR adaptor ligation, DpnII digestion, and PCR amplification were performed as described. DNA was sonicated using a Diagenode Bioruptor Pico for 6 cycles (30 s on/90 s off at 4 °C), and DNA fragments were analyzed using an Agilent Bioanalyzer to confirm ∼300 bp fragment size. DamID adaptor removal and DNA cleanup were performed as previously described, and samples were submitted to BGI Genomics for library construction and sequencing. Libraries were prepared at BGI Genomics following a ChIP-seq workflow and sequenced on the NovaSeq S4.

TaDa sequencing data were processed using the Damsel pipeline (Page et al. 2024). Reads were aligned to the *Drosophila melanogaster* dm6 genome and counted over GATC fragments using countBamInGATC. A single shared Dam-only control was used across all genotypes (*Kdm5^WT^*, *Kdm5^A224T^*, and Kdm5^JmjC*^). Differential binding was performed using edgeR at the GATC-fragment level, with genotype contrasts assessed relative to the shared Dam control (Robinson et al. 2010). Wild-type peaks were defined from the Dam:KDM5^WT^ vs Dam comparison using Damsel. GATC-fragment-level statistics were mapped onto KDM5^WT^ peaks by genomic overlap, and one representative region per peak was retained based on the lowest FDR. For gene-level analyses, peaks were annotated to nearby genes, and a single value per gene was obtained by selecting the peak with the lowest FDR. Significance was defined as FDR < 0.05. Global binding changes were assessed by examining the distributions of log2 fold changes across all KDM5^WT^-defined peaks.

### TurboID-mediated biotinylated protein enrichment

Data from TurboID-KDM5^WT^ (N-terminally tagged KDM5), *w^1118^*, and TurboID-dCas9 have been previously published (Yheskel et al. 2023). Importantly, the experiments described here were carried out concurrently with those reported in previously published data. 2- to 5-d-old flies of the genotypes *Kdm5^Δ^*, Ubi-Gal4/*Kdm5^Δ^* + ; UASp-Kdm5^WT or variant^ were flash-frozen in liquid nitrogen, vortexed, and size-selected for heads. Processing and analyses were carried out similarly to our prior study (Yheskel et al. 2023). Using 10 heads per replicate, samples were homogenized in 250 µL RIPA buffer (Thermo Fisher Scientific, 89901) supplemented with Halt Protease Inhibitor Cocktail (Thermo Fisher Scientific, 78430) and centrifuged at 4 °C for 10 min at 15,000XG to remove debris. 100 µL of Pierce Streptavidin Magnetic Beads (Thermofisher, 88817) were washed twice with RIPA, and the cleared lysate was added. The lysate-bead mixture was incubated with rotation at 4 °C overnight. The next day, the lysate was discarded, and the beads were washed twice with RIPA, once with 1 M KCl, once with 0.1 M Na2HCO3, once with 1 M Urea in 10 mM Tris, pH 8.0, and twice again with RIPA. For Western Blot analyses all RIPA was removed and biotinylated proteins were eluted with 4× NuPAGE LDS Sample Buffer (Invitrogen, NP0007) supplemented with 2 mM biotin and 20 mM DTT.

Proteins were digested directly on streptavidin beads. 5 mM DTT and 50 mM ammonium bicarbonate (pH 8) were added to the solution, which was left at room temperature for about 1 h to reduce disulfide bonds. Samples were then alkylated with 20 mM iodoacetamide in the dark for 30 min. Afterward, 500 ng of trypsin was added to the samples, which were digested at 37 °C for 18 h. The peptide solution was dried in a vacuum centrifuge. Prior to mass spectrometry analysis, samples were desalted using a 96-well plate filter (Orochem) packed with 1 mg of Oasis HLB C-18 resin (Waters). Briefly, the samples were resuspended in 100 µL of 0.1% TFA and loaded onto the HLB resin, which was previously equilibrated using 100 µL of the same buffer. After washing with 100 µL of 0.1% TFA, the samples were eluted with a buffer containing 70 µL of 60% acetonitrile and 0.1% TFA and then dried in a vacuum centrifuge.

Samples were resuspended in 10 µL of 0.1% TFA and loaded onto a Dionex RSLC Ultimate 300 (Thermo Scientific), coupled online with an Orbitrap Fusion Lumos (Thermo Scientific). Chromatographic separation was performed with a 2-column system, consisting of a C-18 trap cartridge (300 µm ID, 5 mm length) and a picofrit analytical column (75 µm ID, 25 cm length) packed in-house with reversed-phase Repro-Sil Pur C18-AQ 3 µm resin. Peptides were separated using a 90 min gradient from 4 to 30% buffer B (buffer A: 0.1% formic acid, buffer B: 80% acetonitrile + 0.1% formic acid) at a flow rate of 300 nL/min. The mass spectrometer was set to acquire spectra in a data-dependent acquisition (DDA) mode. Briefly, the full MS scan was set to 300 to 1,200 m/z in the orbitrap with a resolution of 120,000 (at 200 m/z) and an AGC target of 5 × 10e5. MS/MS was performed in the ion trap using the top speed mode (2 s), an AGC target of 1 × 10e4 and an HCD collision energy of 35. Raw files were searched using Proteome Discoverer software (v2.4, Thermo Scientific) using SEQUEST search engine and the UniProt database of *Drosophila melanogaster*. The search for the total proteome included variable modification of N-terminal acetylation and fixed modification of carbamidomethyl cysteine. Trypsin was specified as the digestive enzyme with up to 2 missed cleavages allowed. Mass tolerance was set to 10 ppm for precursor ions and 0.2 Da for product ions. Peptide and protein false discovery rate was set to 1%. Following the search, data were processed as described by Aguilan et al. (Aguilan et al. 2020). Briefly, proteins were log2 transformed, normalized by the average value of each sample and missing values were imputed using a normal distribution 2 standard deviations lower than the mean. Statistical regulation was assessed by first performing a Fisher's F-Test to evaluate the equality of variance at a given protein between genotypes. If the variances were statistically unequal, differences were assessed using a Welch's heteroscedastic *T*-test (*F*-test *P*-value < 0.05), otherwise a standard *T*-test was applied. Data were assumed to be Gaussian distributed.

### H3K4me3 CUT&RUN

To quantify H3K4me3 and accessibility, we performed CUT&RUN as in our prior analysis of Kdm5^JmjC*^ and *Kdm5^L854F^* (Yheskel et al. 2025). Brains from 50 adult flies (2 to 5 d old) were put in 1 mL of ice-cold 1X PBS and centrifuged at 4 °C for 5 min at 1000XG. PBS was removed, and the cell pellet was resuspended in 150 μL of 100 mg/mL collagenase, type I (Thermo Fisher, 17018029), and incubated at 25 °C (shaking at 500 rpm) for 2 h. To facilitate digestion, the suspension was vigorously pipetted up and down 20 times every 10 to 15 min. Suspensions were then centrifuged at 4 °C for 5 min at 1000XG and resuspended in PBS + 10% DMSO. Cell number was quantified using a hemocytometer. The remaining 100 μL was slowly frozen in a Mr. Frosty Freezing Container (Thermo Fisher, 5100-0001) at −80 °C.

For CUT&RUN, the Epicypher CUTANA Kit (Epicypher, 14-1048) and Epicypher Library Prep Kit (Epicypher, 14-1001) were used as directed using the single-cell suspensions generated. The anti-H3K4me3 antibody used was Epicypher Cat# 13-0041 (RRID:AB_3076423), and IgG (Epicypher Cat# 13-0042, RRID:AB_2923178). Samples were sequenced on the Illumina MiSeq Platform. All samples were normalized to spike-in *E. coli* DNA. Samples were sequenced on the Illumina MiSeq Platform.

Raw reads were quality-controlled and trimmed using rfastp (v1.6.0), and aligned to the dm6 *Drosophila melanogaster* genome assembly using Rsubread (2.10.5) (Chen et al. 2018; Liao et al. 2019). BAM files were normalized by dividing the number of *E.coli* reads in each sample by the lowest number of *E. coli* reads across samples. Samtools (v1.14) was then used to scale BAM files by this normalization factor using (Samtools view -s (normalization factor)) (Li et al. 2009). BigWigs were generated using the deepTools (v3.5.1) bamCoverage function (Ramírez et al. 2014). Profiles and heatmaps were generated using the deepTools computeMatrix and plotProfile functions. Peaks were called using Genrich(v0.6.1) and DiffBind (v3.6.1) was used to perform differential peak analyses (Ross-Innes et al. 2012). Reads in each peak were counted using dba.count(summits = FALSE). ChIPSeeker (v1.32.0) was used to perform peak annotation, and clusterProfiler(v4.4.4) was used to perform Gene Ontology Analyses (Yu et al. 2012; Wang et al. 2022).

### Published datasets used in this study

Kdm5^JmjC*^ and *Kdm5^A224T^* RNA-seq data derived from adult heads have been previously published and are available at GEO:GSE100578 and GEO:GSE245380 (Zamurrad et al. 2018; Yheskel et al. 2024).

## Results

### KDM5^A224T^ is not predicted to dramatically alter the structure of KDM5

The KDM5^A224T^ variant affects a residue near the ARID motif that is conserved across human KDM5A, KDM5B, KDM5C, and KDM5D, as well as in *Drosophila* KDM5, suggesting its importance for an activity shared by all paralogs (Fig. 1, a and b). One function likely to be altered is the ability of the variant KDM5^A224T^ protein to demethylate H3K4me3 (Yheskel et al. 2024). In keeping with this, the AlphaFold-predicted position of the A224 residue within the model of the wild-type protein places it in proximity to the enzymatic JmjC domain, although not to the catalytic pocket (Fig. 1c) (Jumper et al. 2021). Aligning the predicted structures of KDM5^WT^, KDM5^JmjC*^, and KDM5^A224T^ revealed near-identical overlap, except for the intrinsically disordered C-terminal region, which is inherently difficult to model (Fig. 1, d and e). The AlphaFold models therefore predict little overall structural difference between KDM5^WT^ and KDM5^A224T^, although the effects of missense variants can be difficult to accurately detect (Buel and Walters 2022). To further examine potential local structural effects of the A224T change, we compared hydrogen–bond interactions surrounding residue 224 in the KDM5^WT^ and KDM5^A224T^ models using ChimeraX (Pettersen et al. 2004, 2021). Substituting alanine with threonine at this position is predicted to introduce an additional hydrogen bond with isoleucine at position 693 within the JmjC domain (Fig. 1, f and g). This has the potential to alter local structural constraints and impact enzymatic function while preserving the overall protein structure. To examine structural differences more broadly, we examined 5 independent AlphaFold models for each genotype to account for variability in model generation. These were used to generate residue interaction networks (RINs) using RING to assess how intramolecular interactions might change across models and genotypes (Del Conte et al. 2024). RINs were highly correlated for KDM5^A224T^ and KDM5^JmjC*^, with only a small number of edges gained or lost, suggesting very little change to the overall network of intramolecular interactions in these structures (Supplementary Fig. 1, a and b). This was also true within 8 Å of the catalytic pocket, with over 90% of the edges required for enzymatic activity expected to be maintained in both KDM5^JmjC*^ and KDM5^A224T^ (Supplementary Fig. 1, c and d). There were, however, a small number of intramolecular interactions that were gained or lost in KDM5^JmjC*^ or KDM5^A224T^, some of which were shared, suggesting that the 2 variant proteins have overlapping and distinct features (Supplementary Fig. 1e). Overall, the AlphaFold models predict relatively minor structural changes in KDM5^JmjC*^ and KDM5^A224T^, suggesting that functional deficits are unlikely to result from large-scale conformational changes to these variant proteins.

**Fig. 1.**
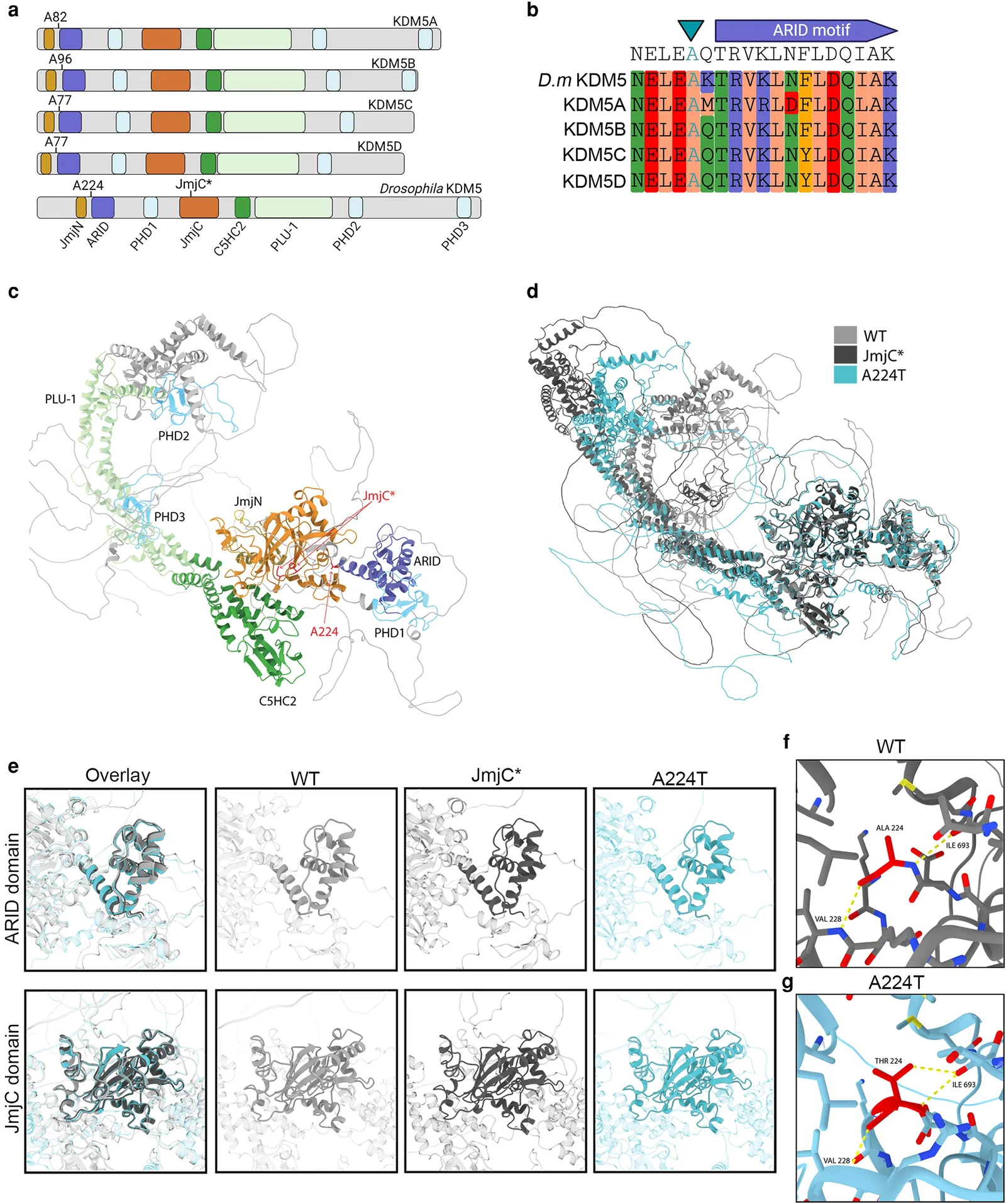
Modeling the KDM5^A224T^ variant protein suggests that it is likely to be structurally similar to wild-type KDM5. a) Schematic showing the domains of human KDM5A, KDM5B, KDM5C, and KDM5D in addition to the position of the ID-associated change identified in KDM5C (A77T) in all paralogs. *Drosophila* KDM5 additionally shows the position of the JmjC* allele, which alters histidine 637 and glutamic acid 639 within the JmjC domain to alanine. b) Alignment of *Drosophila* KDM5 and human KDM5A, KDM5B, KDM5C, and KDM5D, showing the region surrounding the pathogenic alanine to threonine change (indicated by arrowhead). The beginning of the ARID region of KDM5 proteins based on InterPro is indicated above. c) AlphaFold model of *Drosophila* KDM5 showing the position of the 2 changes that abolish enzymatic activity in KDM5^JmjC*^ in addition to A224 (equivalent to A77 in KDM5C) in red. Domain colors match those used in panel A. d) Comparison of AlphaFold-generated models of KDM5^WT^, KDM5^A224T^ KDM5^JmjC*^. e) Comparison AlphaFold-generated models of KDM5^WT^, KDM5^A224T^ KDM5^JmjC*^, focusing on the ARID motif (top) and JmjC (bottom) domains. f) Hydrogen-bonding interactions detected in the region of A224 of wild-type KDM5 using the FindHBond tool in ChimeraX. g) Hydrogen-bonding interactions detected in KDM5^A224T^ using the FindHBond tool in ChimeraX.

### KDM5^A224T^ causes H3K4me3 changes similar to those induced by the demethylase-dead KDM5^JmjC*^ allele

Despite the predicted structural similarity of the catalytic pocket in KDM5^A224T^ to that of KDM5^WT^, this variant protein clearly exhibits reduced ability to demethylate H3K4me3 (Yheskel et al. 2024). To quantify the effects of *Kdm5^A224T^* on H3K4me3 in the adult brain, we used Cleavage Under Targets & Release Using Nuclease (CUT&RUN) and compared this with published data from *Kdm5^WT^* and Kdm5^JmjC*^ (Skene and Henikoff 2017; Yheskel et al. 2025). To limit our analysis to regions of the genome occupied by KDM5, which we have found to largely colocalize with the presence of H3K4me3, we incorporated published KDM5 ChIP-seq data derived from whole adult flies (Fig. 2a) (Liu and Secombe 2015; Yheskel et al. 2025). This revealed that *Kdm5^A224T^* and Kdm5^JmjC*^ showed a similar expanded distribution of H3K4me3 (Fig. 2, a and b; compare black and teal lines to the control gray signal). To identify genes associated with altered H3K4me3 in *Kdm5^A224T^* animals, we used Diffbind to identify genomic regions with significant changes in H3K4me3, and then ChIPseeker to assign them to the nearest gene (Ross-Innes et al. 2012). 1,505 genes showed significantly increased H3K4me3, while only 3 showed reduced signal (Fig. 2, c and d; |log2FC| > 1 and FDR < 0.01; Supplementary file 1). The changes to H3K4me3 in *Kdm5^A224T^* were strikingly similar to those seen in Kdm5^JmjC*^ animals, suggesting that KDM5^A224T^ behaves functionally as a demethylase dead protein (*r* = 0.97, *P* < 1e−16; Fig. 2d). We previously observed in our studies of Kdm5^JmjC*^ that increased H3K4me3 does not correlate with altered gene expression seen by RNA-seq analyses (Yheskel et al. 2024). Similarly, altered H3K4me3 in *Kdm5^A224T^* did not predict altered mRNA levels, with most genes showing unchanged expression (Fig. 2e). Changes in H3K4me3 alone at KDM5-bound genes are therefore insufficient to drive the observed changes to overall gene expression.

**Fig. 2.**
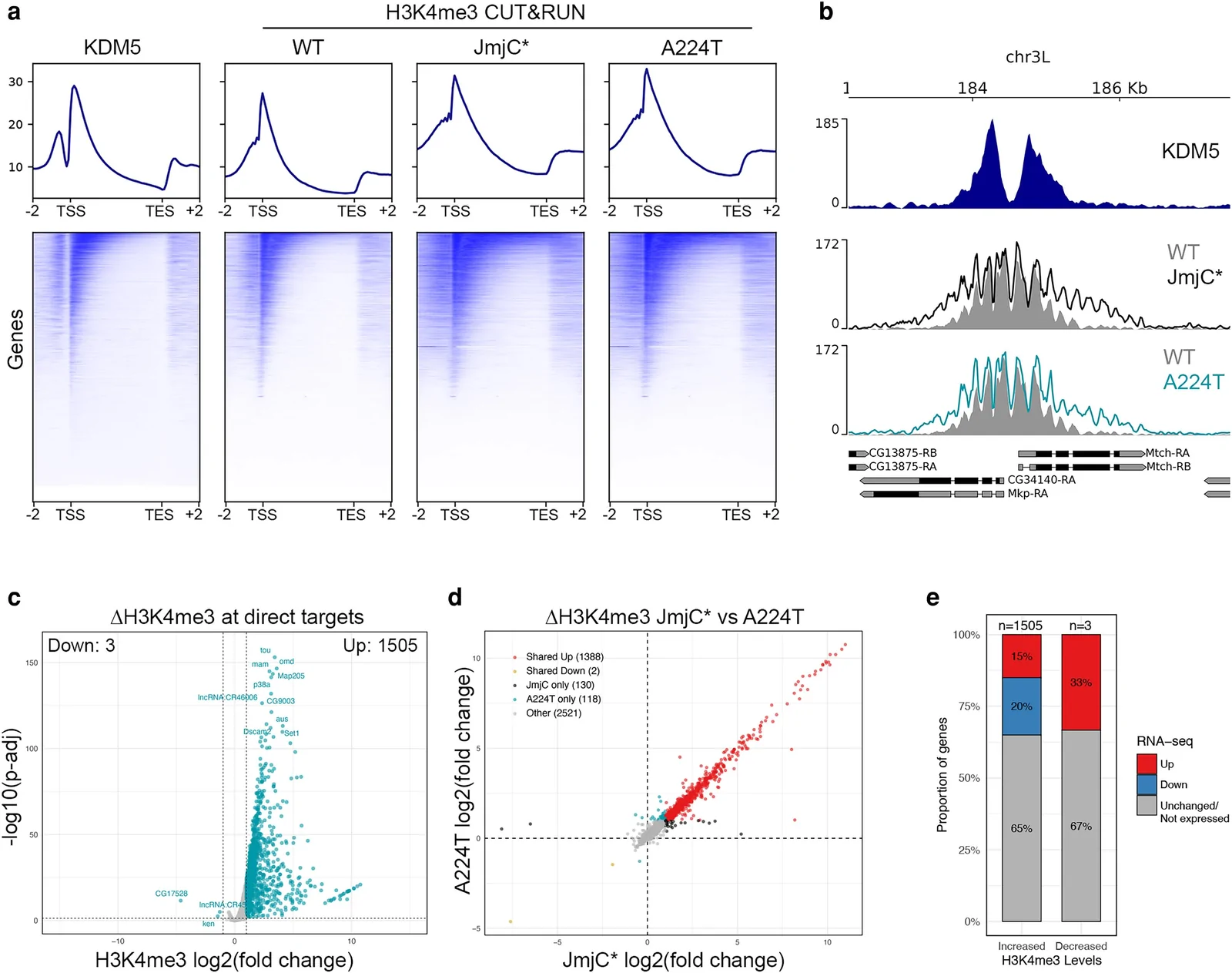
H3K4me3 is similarly increased in the brains of *Kdm5^A224T^* and Kdm5^JmjC*^ animals. a) Profiles and heatmaps showing the distribution of KDM5 (ChIP-seq data from whole wildtype adults) and H3K4me3 CUT&RUN from *Kdm5^WT^*, Kdm5^JmjC*^, and *Kdm5^A224T^* relative to the transcriptional start site (TSS) and transcriptional end site (TES) across all genes. b) Genome track showing KDM5 ChIP-seq and H3K4me3 CUT&RUN from *Kdm5^WT^* compared to Kdm5^JmjC*^ and *Kdm5^A224T^*. Genes are shown below the traces. c) Volcano plot showing changes to H3K4me3 (Diffbind analysis) in *Kdm5^A224T^* at KDM5-bound genes (ChIP-seq data). Dots in the upper rightmost section indicate genes with significantly altered H3K4me3 (|log2FC| > 1 and FDR < 0.01). d) Scatterplot showing correlation between changes to H3K4me3 for KDM5-bound genes from Kdm5^JmjC*^ (*x*-axis) and *Kdm5^A224T^* (*y*-axis). Gene numbers for each category are indicated in the legend. e) Stacked bar graph separating genes with significantly increased (left) or decreased (right) H3K4me3 in *Kdm5^A224T^* and the proportion of those genes that show increased, decreased, and unchanged/unexpressed genes in gray based on RNA-seq data (adult head). The number of genes in each category is indicated by *n*.

### *Kdm5^A224T^* and Kdm5^JmjC*^ affect shared and unique gene expression programs

Although KDM5^JmjC*^ and KDM5^A224T^ both lack the ability to demethylate H3K4me3, it is likely that the A224T change also interferes with one or more other KDM5 activities. This is based on analyses of neuroanatomical changes in mushroom body neurons, which mediate many aspects of cognition in *Drosophila*, where the 2 alleles show different phenotypes (Aso et al. 2014; Zamurrad et al. 2018; Lin 2023). While Kdm5^JmjC*^ animals do not show gross neuromorphological defects, 25% of *Kdm5^A224T^* animals show overgrowth or misprojection of mushroom body neurons that more closely match those caused by loss of KDM5 (Hatch et al. 2021; Yheskel et al. 2024). To gain insights into the transcriptional similarities and differences between Kdm5^JmjC*^ and *Kdm5^A224T^* animals, we re-evaluated previously generated RNA-seq data from adult heads (Zamurrad et al. 2018; Yheskel et al. 2024). This revealed that although many mRNA deficits were shared between the 2 variant strains, each genotype also showed unique changes (Fig. 3, a to c).

**Fig. 3.**
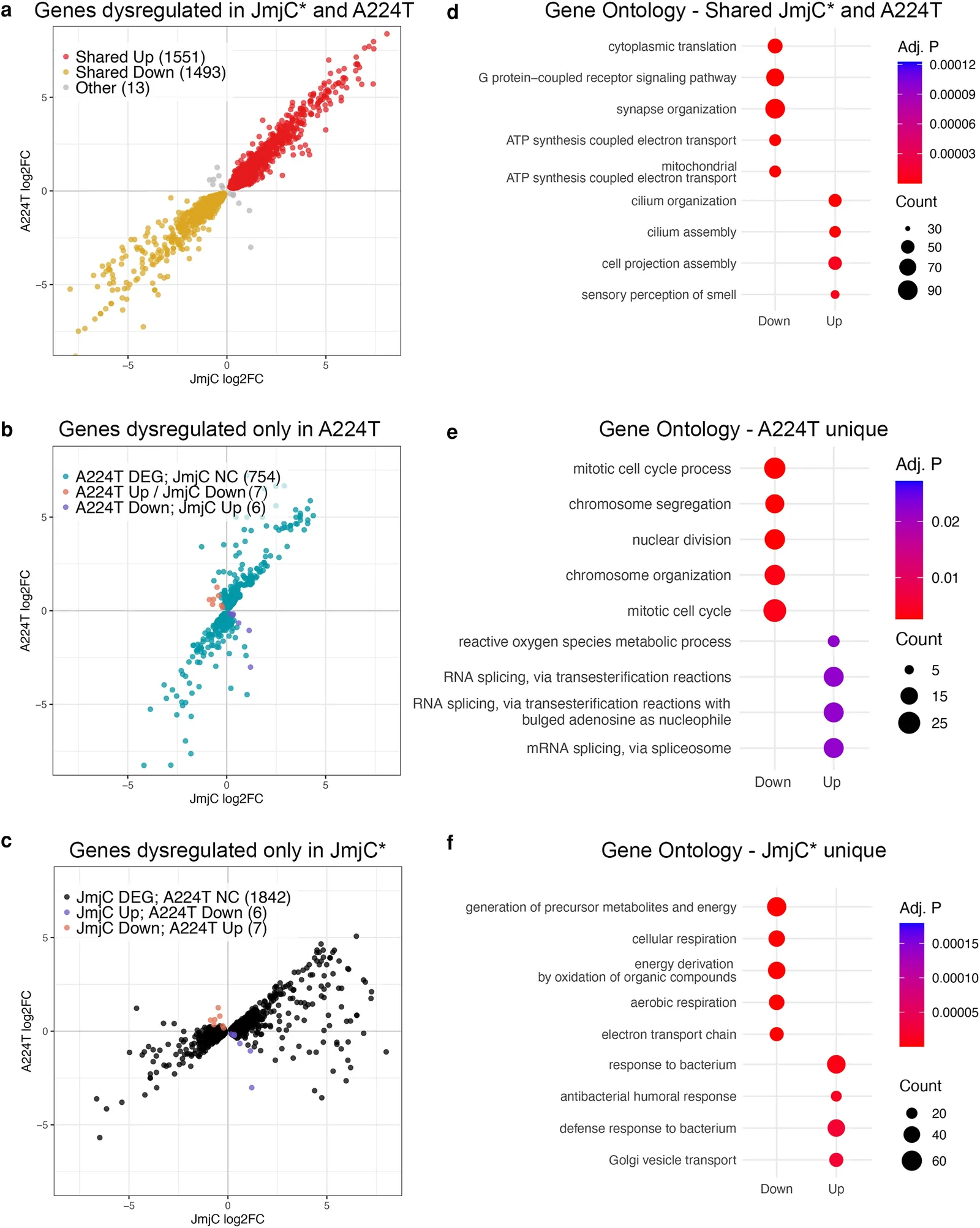
The *Kdm5^A224T^* variant shows similar and distinct transcriptional changes compared to Kdm5^JmjC*^. a) Scatterplot showing the similarity in genes that were dysregulated in *Kdm5^A224T^* and Kdm5^JmjC*^ RNA-seq (FDR < 0.05 cutoff; Pearson correlation r = 0.97, *P* < 1e−16). Genes upregulated in both genotypes are in the upper right quadrant, lower left quadrant are genes downregulated in both, and gray dots show other categories. b) Scatterplot showing genes uniquely dysregulated in *Kdm5^A224T^* RNA-seq. c) Scatterplot showing genes uniquely dysregulated in Kdm5^JmjC*^ RNA-seq. d) Gene ontology (GO) enrichment analysis of up- and downregulated genes that were shared for Kdm5^JmjC*^ and *Kdm5^A224T^*. e) GO enrichment analysis of up- and downregulated genes unique to *Kdm5^A224T^* (unchanged or discordant in Kdm5^JmjC*^). f) GO enrichment analysis of up- and downregulated genes unique to Kdm5^JmjC*^ (unchanged or discordant in *Kdm5^A224T^*).

The 3,057 genes that were dysregulated in both *Kdm5^A224T^* and Kdm5^JmjC*^ were highly correlated and included Gene Ontology (GO) pathways that we have observed to be shared across other ID-allele-containing fly strains (1551 upregulated genes and 1,493 downregulated genes; *r* = 0.97, *P* < 1e−16; Fig. 3a) (Zamurrad et al. 2018; Yheskel et al. 2024). Downregulated genes were enriched for those required for cytoplasmic translation, such as ribosomal protein genes, as well as G protein-coupled receptor signaling pathways, synapse function, and mitochondrial activity (Supplementary file 2; Fig. 3d). Upregulated genes included those involved in cilium organization and function, including components of the Bardet–Biedl syndrome complex that are needed within neurons for transport along microtubules and for propagating signaling cascades (Tian et al. 2023)(Supplementary file 3).

Within the *Kdm5^A224T^* RNA-seq dataset, 767 genes displayed significantly altered expression that were not shared with Kdm5^JmjC*^ (Fig. 3b). The 333 downregulated genes unique to *Kdm5^A224T^* included many with characterized roles in cell division, some of which are additionally known to be important in neuronal development or function. These include genes involved in microtubule organization, such as Pavarotti (pav), Dynein heavy chain (Dhc64C), and Gamma tubulin (γTub23C; Fig. 3e; Supplementary file 4). This suggests that KDM5^A224T^ selectively disrupts a transcriptional program comprising cell cycle-associated genes that also support neuronal structure and function, possibly contributing to the morphological phenotypes observed in these animals. Fewer significantly enriched categories were identified among the 434 upregulated genes unique to *Kdm5^A224T^* (Fig. 3e). These changes were related to antioxidant activity, including peroxiredoxin 3 (Prx 3) and Catalase (Cat), and to RNA-binding proteins implicated in splicing regulation (Supplementary file 5). Interestingly, although the Kdm5^JmjC*^ allele was generated with the intention of specifically disrupting the enzymatic activity of KDM5, there are gene expression changes that are not shared with *Kdm5^A224T^* (1855 genes; Fig. 3c). This suggests that the JmjC* allele might disrupt additional functions of the JmjC domain or other regions of the protein that rely on this motif. These include the downregulation of genes implicated in mitochondrial function and the upregulation of genes related to immunity (Fig. 3f; Supplementary files 6 and 7).

### KDM5^A224T^ displays only minor changes to genomic recruitment that do not correlate with changes to mRNA levels

The A224T change occurs at a residue that is linearly distant from the enzymatic JmjC domain, which spans amino acids 591 to 757 (Blum et al. 2025). Alanine 224 lies at the N-terminus of the ARID motif, which binds DNA in vitro, suggesting that changes to this residue may alter this function in addition to the enzymatic activity of KDM5. In silico and in vitro analyses indicated that human KDM5C^A77T^ does not alter the nucleic acid binding function of the ARID (Scibetta et al. 2007; Tu et al. 2008; Yao et al. 2010; Peng et al. 2015). However, given its proximity to this motif, we assessed the ability of KDM5^A224T^ to be recruited to target genes. To do this, we used Targeted DamID (TaDa), which enables the identification of genomic regions of KDM5 occupancy by local recruitment of a DNA methyltransferase (Marshall et al. 2016; Aughey et al. 2019). This antibody-free method requires small amounts of material, with methylated DNA fragments being purified and sequenced to assess the behavior of Dam:KDM5^WT or variant^ fusion proteins.

For TaDa, we expressed Dam:KDM5^WT^, Dam:KDM5^A224T^ in 3 to 5-d-old *Kdm5^WT^* or *Kdm5^A224T^* flies, respectively. As a control, Dam alone was expressed in age-matched wild-type flies. Whole heads were used across all conditions. Data were analyzed using Damsel with edgeR, which enables replicate-aware statistical comparisons between *Kdm5^WT^* and *Kdm5^A224T^* TaDa samples (Robinson et al. 2010; Page et al. 2024) (Supplementary file 8). Comparing KDM5^A224T^ to KDM5^WT^ revealed similar overall recruitment profiles, suggesting that the widespread increase in H3K4me3 observed in *Kdm5^A224T^* was unlikely to be caused by altered recruitment (Fig. 4a). However, some significant differences were observed in the distribution of KDM5^A224T^, with 32 of the 2466 peaks defined for KDM5^WT^ being decreased (1.3%) and 8 being increased (0.3%) (FDR < 0.05, |Log2FC| > 0.5; Fig. 4b; Supplementary file 8). Based on our observation that *Kdm5^A224T^* behaves as a demethylase-dead allele, we compared this with previously published TaDa data using Kdm5^JmjC*^, which also showed a small number of changes in genomic recruitment (Yheskel et al. 2025). Direct comparison of KDM5^A224T^ and KDM5^JmjC*^ revealed more differences than were observed for either genotype relative to KDM5^WT^ (162 changes), consistent with KDM5^A224T^ having defects beyond loss of demethylase activity (Fig. 4c) (Yheskel et al. 2025). To capture genome-wide trends, we analyzed the distribution of log2 fold changes across the 2 mutant genotypes and observed small directional shifts in occupancy, with KDM5^A224T^ showing a slight increase and KDM5^JmjC*^ a slight decrease relative to KDM5^WT^ (Supplementary Fig. 2, a and b). We also analyzed our data for peaks detected in KDM5^A224T^ TaDa but not in KDM5^WT^. This revealed 168 new peaks that were distributed among intergenic and intronic regions and, to a lesser extent, promoters (Fig. 4d; Supplementary file 8). Some of these additional genomic recruitment events were likely due to the loss of enzymatic activity in KDM5^A224T^, as 31 overlapped with new peaks detected in KDM5^JmjC*^ TaDa (Fig. 4e). To determine whether TaDa-detected changes in KDM5^A224T^ were likely to contribute to changes in gene expression, we integrated TaDa with RNA-seq data. Overwhelmingly, changes to KDM5^A224T^ recruitment occurred at genes that had no detectable expression or showed unchanged mRNA levels in *Kdm5^A224T^*, although a small number of changes were seen at genes with altered expression (Supplementary Fig. 2c; Supplementary file 8). Thus, although some changes in KDM5^A224T^ recruitment were observed, it remains unclear whether these differences contribute to the observed phenotypes.

**Fig. 4.**
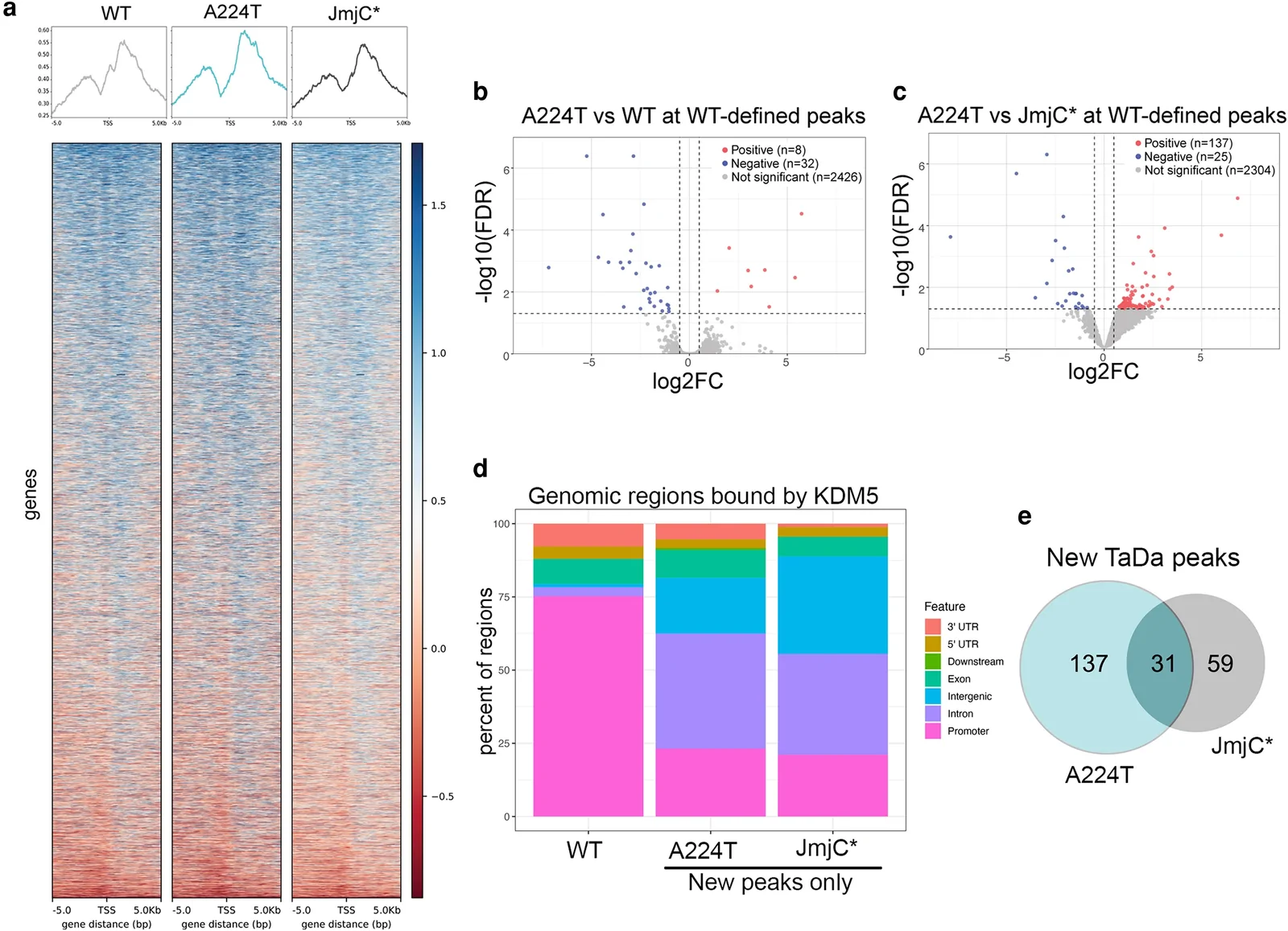
Changes to gene expression and H3K4me3 in *Kdm5^A224T^* are not explained by altered KDM5 recruitment. a) Metaplots and heatmaps showing DamID log2(Target/Dam) at KDM5^WT^-occupied transcriptional start sites (TSSs). b) Volcano plots showing gained and lost KDM5^WT^-defined peaks for *Kdm5^A224T^* vs *Kdm5^WT^*, with significant peaks defined as FDR < 0.05 and |log2FC| ≥ 0.5. Upregulated peaks are shown in the upper rightmost section and downregulated in the upper leftmost section. c) Volcano plots showing gained and lost KDM5^WT^-defined peaks for *Kdm5^A224T^* vs Kdm5^JmjC*^, with significant peaks defined as FDR < 0.05 and |log2FC| ≥ 0.5. peaks upregulated in *Kdm5^A224T^* are shown in the upper rightmost section and downregulated in the upper leftmost section. d) Stacked bar graph showing the proportion of peaks located in different genomic regions. KDM5^WT^ shows all peaks from wild-type animals. KDM5^A224T^ and KDM5^JmjC*^ show new peaks that were not detected in wild-type animals. e) Venn diagram showing overlap in the new peaks identified in KDM5^A224T^ and KDM5^JmjC*^.

### KDM5^A224T^ alters the proximitome of KDM5

In addition to altering enzymatic function, in silico predictions suggest that the N-terminal region of the ARID encompassing the KDM5C^A77T^ variant might mediate intramolecular interactions with other KDM5 domains or intermolecular interactions with other proteins (Peng et al. 2015). To examine this possibility, we used the TurboID proximity-mediated biotinylation system, which we previously used to identify candidate KDM5-interacting proteins in the adult brain (Yheskel et al. 2023). In addition to generating transgenic flies able to express an N-terminal TurboID fusion of KDM5^A224T^, we created strains able to express KDM5^JmjC*^ to compare the behavior of this variant (UASp-*TurboID*:*Kdm5^A224T^* and UASp-*TurboID*:Kdm5^JmjC*^). To enhance our ability to detect interactors, we created flies in which the sole source of KDM5 was TurboID:KDM5^WT or variant^ by ubiquitously expressing fusion proteins at approximately endogenous levels in a *Kdm5^Δ^* background using Ubi-Gal4, similar to our prior study of wild-type KDM5 (Fig. 5, a to d) (Yheskel et al. 2023). The ability of the TurboID:KDM5^A224T^ and TurboID:KDM5^JmjC*^ to rescue the lethality of the *Kdm5^Δ^* allele demonstrates the functionality of these fusion proteins (Fig. 5, b to d). Adult heads were used to purify biotinylated proteins, which were identified using liquid chromatography-tandem mass spectrometry (LC-MS/MS; Supplementary file 9). Principal component analyses of the samples from *Kdm5^WT^*, *Kdm5^A224T^*, and Kdm5^JmjC*^ animals show clear separation of the genotypes, with *Kdm5^A224T^* being the most distinct (Fig. 5e). To assess how interactions might be altered by the 2 KDM5 variants, we examined 87 proteins identified as high-confidence KDM5 TurboID interactors, defined by their reproducible enrichment across biological replicates relative to untagged and dCas9:TurboID controls (Yheskel et al. 2023). This was possible because the TurboID studies of KDM5^A224T^ and KDM5^JmjC*^ were conducted concurrently with these original experiments. To exclude changes in proximity caused by altered expression levels in *Kdm5^A224T^* or Kdm5^JmjC*^, we eliminated those with decreased mRNA levels from our analyses (|Log2FC| > 1, FDR < 0.05; Supplementary file 9).

**Fig. 5.**
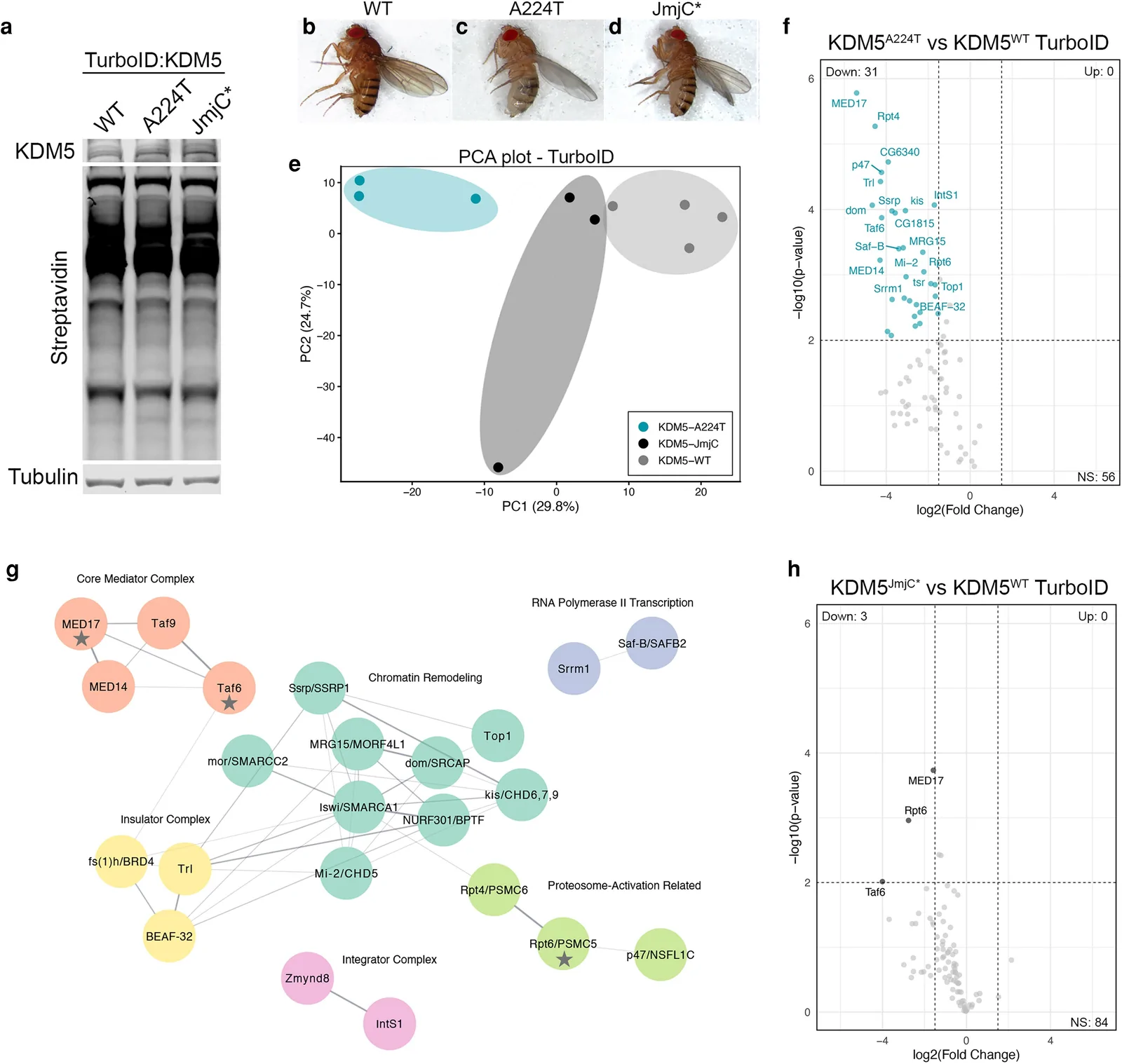
TurboID-detected proximal proteome changes caused by KDM5^A224T^ and KDM5^JmjC*^. a) Western blot showing expression of KDM5 (anti-KDM5), level of biotinylation via a streptavidin-680 conjugate, and loading control Tubulin in animals of the genotypes *Kdm5^Δ^*/*Kdm5^Δ^*, Ubi-Gal4; UASp-TurboID:KDM5^WT^ (WT), *Kdm5^Δ^*/*Kdm5^Δ^*, Ubi-Gal4; UASp-TurboID:KDM5^A224T^ (A224T), *Kdm5^Δ^*/*Kdm5^Δ^*, Ubi-Gal4 ; UASp-TurboID:KDM5^JmjC*^ (JmjC*). b) Ubiquitous expression of wild-type KDM5 as TurboID fusion produces adult flies that are morphologically normal. Genotype is *Kdm5^Δ^*/*Kdm5^Δ^*, Ubi-Gal4; UASp-TurboID:KDM5^WT^ (WT). Female fly shown, although males behaved similarly. c) Ubiquitous expression of TurboID:KDM5^A224T^ produces adult flies, although at a reduced frequency compared to WT. Genotype is *Kdm5^Δ^*/*Kdm5^Δ^*, Ubi-Gal4 ; UASp-TurboID:KDM5^A224T^ (A224T). Female fly shown, although males behaved similarly. d) Ubiquitous expression of TurboID:KDM5^JmjC*^ produces adult flies, although at a reduced frequency compared to WT. Genotype is *Kdm5^Δ^*/*Kdm5^Δ^*, Ubi-Gal4 ; UASp-TurboID:KDM5^JmjC*^ (JmjC*). Female fly shown, although males behaved similarly. e) Principal component analysis plot showing the relationship between the quadruplicate TurboID:KDM5^WT^ and triplicate datasets of TurboID:KDM5^A224T^ and TurboID:KDM5^JmjC*^. f) Volcano plot showing altered proximity of 87 high confidence interactors in TurboID:KDM5^A224T^ compared to TurboID:KDM5^WT^. 31 proteins were significantly reduced using *P*-value < 0.01, |log2FC| > 1.5. g) String-db guided clustering of the proteins significantly affected by TurboID:KDM5^A224T^. Unconnected proteins were excluded. Stars indicate proteins that were also significantly down in TurboID:KDM5^JmjC*^. h) Volcano plot showing altered proximity of 87 high confidence interactors in TurboID:KDM5^JmjC*^ compared to TurboID:KDM5^WT^. 3 proteins were significantly reduced using *P*-value < 0.01, |log2FC| > 1.5. Also indicated with stars in panel G.

Comparing KDM5^A224T^ to KDM5^WT^ revealed reduced proximity to 31 proteins using stringent cutoffs of *P* < 0.01 and |log2FC| > 1.5 (Figure 5f; Supplementary file 9). These included 2 of the 3 components of the Mediator complex identified in our KDM5^WT^ TurboID study that perform key scaffold functions (MED14 and MED17), as well as 2 members of TFIID (Taf4 and Taf9; Fig. 5g) (Richter et al. 2022). In addition, proteins with connections to the chromatin remodeling Imitation Switch (ISWI) protein that is the ATP-dependent enzyme at the core of several different chromatin remodeling complexes (eg NURF, CHRAC, ACF) were reduced (Fig. 5g) (Eustermann et al. 2024; Wang et al. 2026). One possibility is that the KDM5^A224T^ protein may lead to altered gene expression, at least in part through a reduced ability to coordinate events related to accessibility and preinitiation complex assembly at promoter regions. In contrast, KDM5^JmjC*^ had only 3 proteins with reduced proximity, all of which were also observed in KDM5^A224T^: the mediator complex component MED17, the TFIID component Taf6, and the proteasome component Rpt6 (Fig. 5h). These data suggest that, while the primary defect of KDM5^JmjC*^ is the loss of enzymatic function, the JmjC domain likely has additional activities that may or may not be directly related to its enzymatic activity (Fig. 5g).

## Discussion

Here, we use a pathogenic *KDM5C* variant to dissect the gene regulatory functions of KDM5 proteins. The *KDM5C^A77T^* allele was observed to segregate in a family with X-linked ID and is associated with severe cognitive deficits, seizures, spasticity, aggression, and related traits in hemizygous males, and milder symptoms in heterozygous females (Abidi et al. 2008; Simensen et al. 2012; Grafodatskaya et al. 2013). Despite considerable clinical descriptions, little is known about the molecular deficits associated with KDM5C^A77T^, which occurs adjacent to the ARID and is conserved in all other KDM5 family proteins in humans as well as across species. Consistent with a conserved role for this residue in neural function, *Kdm5^A224T^* animals exhibit learning and memory defects, seizure susceptibility, and altered locomotion (Yheskel et al. 2024). Analyses shown here further demonstrate that KDM5^A224T^ disrupts both enzymatic and non-enzymatic activities of KDM5, revealing that these functions are closely integrated yet at least partially separable. Importantly, we find that KDM5^A224T^ impairs proximity to several gene-regulatory complexes, suggesting that altered complex assembly or stability may contribute to features associated with this pathogenic protein.

Quantifying H3K4me3 levels in the brains of *Kdm5^A224T^* animals revealed that this variant behaves functionally as a demethylase-dead allele. Precisely why this amino acid substitution abolishes catalytic activity remains unclear. Current structural models do not predict that the KDM5^A224T^ protein disrupts the overall protein architecture or the catalytic pocket. Instead, the substitution is predicted to introduce an additional hydrogen bond between T224 and I693 within the JmjC domain, which could subtly alter the conformational dynamics required for efficient H3K4me3 demethylation. This interpretation is supported by comparison with another ID-associated missense variant, *KDM5C^D87G^*, located only 10 residues away. Although KDM5C^D87G^ is associated with a similar spectrum of cognitive and behavioral traits, it retains histone demethylase activity in vitro, and in vivo studies of the analogous allele in *Drosophila, Kdm5^D234G^*, show wild-type levels of bulk H3K4me3 (Tahiliani et al. 2007; Yheskel et al. 2024). Unlike A224T, the predicted hydrogen-bonding network surrounding D234 is preserved upon substitution with glycine, with interactions to K230 and K238 remaining intact. Subtle changes in local structural dynamics caused by missense changes might lead to distinct consequences for KDM5 catalytic activity.

Our occupancy data further indicate that the loss of H3K4me3 regulation in KDM5^A224T^ is not caused by defective promoter recruitment. Despite the predicted function of the ARID region in nucleic acid binding, we observed relatively few changes in KDM5^A224T^ genomic recruitment, and these do not account for the widespread increase in promoter-proximal H3K4me3 or the observed changes in gene expression programs. These findings suggest that the A224T substitution disrupts KDM5 catalytic function after chromatin binding. Future work defining the molecular mechanism underlying this defect will provide important insights into both KDM5 catalytic regulation and the pathogenesis of KDM5-associated neurodevelopmental disorders.

In contrast to their nearly identical changes to H3K4me3, prior and current data show that *Kdm5^A224T^* and Kdm5^JmjC*^ exhibit both shared and unique transcriptional consequences (Yheskel et al. 2024). This is in keeping with *Kdm5^A224T^* animals exhibiting neuromorphological phenotypes in the adult brain that are absent in Kdm5^JmjC*^ flies, suggesting that the A224 region imparts additional non-enzymatic activities (Zamurrad et al. 2018; Yheskel et al. 2024). The enrichment of genes linked to cytoskeletal organization and cell division among *Kdm5^A224T^*-specific downregulated genes may provide a molecular explanation for the axonal growth and guidance phenotypes observed in this allele but absent in Kdm5^JmjC*^ (Zamurrad et al. 2018; Yheskel et al. 2024). Our data are consistent with a model in which KDM5 proteins act as scaffolds or organizing hubs whose biological functions extend beyond demethylation. Altered protein interactions may contribute to the non-enzymatic transcriptional functions of KDM5 disrupted by KDM5^A224T^. Accordingly, TurboID analyses revealed altered proximity to components of the Mediator and TFIID complexes, as well as those involved in chromatin remodeling, suggesting that KDM5^A224T^ compromises the ability of KDM5 to coordinate multiple transcriptional regulatory complexes at target promoters (Quevedo et al. 2019; Richter et al. 2022; Ramasamy et al. 2023). In contrast, KDM5^JmjC*^ showed significantly altered proximity to only 3 proteins, all of which were also altered with KDM5^A224T^. This included the Mediator component MED17 and the TFIID protein Taf6, although it is notable that the MED17-interacting protein MED14 showed a reduction in proximity that did not quite meet our stringent cutoff (log2FC = −4.3, *P*-value = 0.04). Both KDM5^A224T^ and KDM5^JmjC*^ may therefore affect interactions with Mediator and/or TFIID, which carry out critical functions related to enhancer and promoter activity (Richter et al. 2022). These shared changes in the proximitome raise the possibility that the histone demethylase activity of KDM5 might be mechanistically coupled with complex assembly at promoters. Alternatively, it may reflect changes in proximity resulting indirectly from transcriptional changes, many of which are shared between *Kdm5^A224T^* and Kdm5^JmjC*^ animals. In either case, our findings challenge a simple model in which catalytic and non-catalytic functions operate independently, instead suggesting that interdependent features of KDM5 are needed to regulate gene expression.

Many of the TurboID changes unique to KDM5^A224T^ appeared to center on complexes formed with the ISWI chromatin remodeler (Eustermann et al. 2024). In *Drosophila,* KDM5 has not been functionally linked to the regulation of chromatin accessibility or nucleosome positioning. However, knocking down KDM5B in mouse embryonic stem cells leads to altered nucleosome phasing around promoter regions, which correlates with dysregulated gene expression (Kurup et al. 2019). While KDM5B co-purifies with components of chromatin remodeling complexes (eg NuRD and NURF), how it directly or indirectly regulates nucleosome positioning, and its relationship to its demethylase activity, remains unknown (Nishibuchi et al. 2014; Pavlenko et al. 2022). It is also unclear whether KDM5 family proteins can alter nucleosome spacing in other cell types, such as those in the brain, which is relevant to their roles in neurodevelopmental disorders. Indeed, it is striking that many of the proteins that show reduced proximity to KDM5^A224T^ and KDM5^JmjC*^ are implicated in intellectual disability. Components of the Mediator complex, including MED14 and MED17, have been linked to global developmental delay and/or intellectual disability (Fazio et al. 2025). Similarly, the human homologs of ISWI, SMARCA1 and SMARCA5, are associated with neurodevelopmental disorders (Goodwin and Picketts 2018; Li et al. 2021; Mirzaa et al. 2025). Our findings support a model in which pathogenic missense variants can disrupt combinations of catalytic and scaffolding functions, yielding phenotypes not predicted solely from loss of enzymatic activity. These results further emphasize the need for additional studies of disorder-associated variants to understand how disruption to KDM5 family protein activity leads to cognitive disorders.

## Supplementary Material

jkag219_Supplementary_Data

## Data Availability

All fly strains generated as part of this study are available upon request. TurboID data are publicly available through ProteomeXchange (project PXD057637). Other data are available under BioProject reference PRJNA1188621 and GEO as follows: H3K4me3 CUT&RUN (GSE282535), Targeted DamID (GSE282537). Supplemental material available at G3 online.
