# Supplementary material for: A KDM5 variant outside the catalytic Jumonji C domain disrupts demethylase activity and alters the proximity interactome
Source: G3 (Bethesda). 2026 Aug 6;16(10):jkag219. doi: 10.1093/g3journal/jkag219 (PMC13643812; doi:10.1093/g3journal/jkag219)
Supplement: Supplementary file 1 [file jkag219_supplementary_data.zip › Supplemental_Material_G3-2026-407129.docx]

**Supplementary Figure 1: The overall structure of KDM5^A224T^ is not predicted to be altered based on AlphaFold modeling**

a. Jaccard similarity matrix showing the correlation between RINs generated for KDM5^WT^, KDM5^A224T^ and KDM5^JmjC*^
b. Edges lost or gained in KDM5^A224T^ and KDM5^JmjC*^ RINs, relative to the edges present in the KDM5^WT^ RIN.
c. Proportion of the edges present in the KDM5^WT^ RIN retained in KDM5^A224T^ and KDM5^JmjC*^, restricting to the catalytic pocket, which is defined here as any atom within 8 Å of any anchor residue.
d. Jaccard similarity between KDM5^WT^ and mutant (KDM5^A224T^ and KDM5^JmjC*^) pocket edge sets.
e. Venn diagram showing the shared and unique interactions gained (top) and lost (bottom) in KDM5^A224T^ and KDM5^JmjC*^.

Alt text: Similarity matrices, bar graphs, and Venn diagrams compare residue interaction networks generated from AlphaFold models of wild-type KDM5, A224T, and JmjC*. The analyses show highly similar residue interaction networks, including within the catalytic pocket, with relatively few unique gained or lost interactions

**Supplementary Figure 2: Changes to binding observed in KDM5^A224T^ by TaDa**

a. Histogram showing the global distribution of *Kdm5^A224T^* vs *Kdm5^WT^* at KDM5^WT^-defined peaks.
b. Histogram showing the global distribution of *Kdm5^JmjC*^* vs *Kdm5^WT^* at KDM5^WT^-defined peaks.
c. Stacked bar graph comparing changes in TaDa to those observed in the RNA-seq data. The total number of genes (N) in each category is indicated.

Alt text: Two histograms show the distribution of genome-wide changes in KDM5 binding in A224T and JmjC* relative to wild-type KDM5, while a stacked bar graph compares changes in KDM5 binding with corresponding changes in gene expression.
